# Organ donation in Saudi Arabia: a profile of public knowledge and behavior from an online survey

**DOI:** 10.3389/fpubh.2026.1748131

**Published:** 2026-01-23

**Authors:** Sami Alobaidi

**Affiliations:** Department of Internal Medicine, University of Jeddah, Jeddah, Saudi Arabia

**Keywords:** behavior, knowledge, organ donation, public, Saudi Arabia

## Abstract

**Background:**

Despite advanced healthcare infrastructure and supportive policy, low donor availability remains the major barrier to organ transplantation in the Kingdom of Saudi Arabia (KSA). Multiple interlinked and complex factors, including psychosocial, cultural, and religious factors, are believed to influence organ donation behavior. The current study explored public knowledge, attitudes, and behavioral intentions toward organ donation in KSA to inform targeted interventions.

**Methods:**

A cross-sectional online survey grounded in the Theory of Planned Behavior was administered nationwide between June and December 2022. Eligible participants were KSA residents aged ≥16 years who could read Arabic and provided consent. A convenience sampling method was used to recruit participants; incomplete responses (<80% of items) were excluded. The instrument assessed knowledge, attitudes, subjective norms, perceived behavioral control, and willingness to register as an organ donor.

**Results:**

Indecision about registering as an organ donor predominated across sociodemographic groups. Females reported higher willingness to register than males (10.4% vs. 8.7%) and lower refusal (10.2% vs. 13.6%), with a significant association (*χ*^2^ = 10.3, *p* = 0.006). Nationality was associated with willingness, with Saudi nationals showing higher willingness (*χ*^2^ = 13.8, *p* < 0.001). Occupation (*χ*^2^ = 20.3, *p* = 0.009) and income (*χ*^2^ = 17.3, *p* = 0.008) were also significantly associated with willingness, with non-working and some higher-income groups more often undecided. Overall, a favorable normative climate and substantial religious endorsement coexisted with gaps in specific knowledge and persistent ambivalence.

**Conclusion:**

In KSA, supportive norms and religious approval are necessary but insufficient to translate positive attitudes into registration behavior. Reducing indecision will likely require sustained, targeted education that addresses misconceptions, clarifies the donation process, and leverages trusted religious and community channels.

## Introduction

Organ transplantation is an important medical intervention that offers a better quality of life and a better prognosis for people suffering from end-stage organ failure. Despite this, the organ donation rate is significantly lower compared to the need, and the most important barrier is the lack of available donors (1, 2). This public health problem is profoundly relevant in the Kingdom of Saudi Arabia (KSA), where the number of available organ donors is relatively low compared to the availability of advanced healthcare facilities and supportive government policies (2, 3). Studies indicate that the gap between donor organ supply and demand in KSA is significantly high, with only 2–4 transplants per million population, which contributes to the ever-increasing waiting lists for donor organs (4, 5). This persistent gap between organ supply and demand highlights an urgent need for context-specific strategies that can be employed by policymakers to improve donor registration and actual organ donation rates.

Among the factors that may help to close this gap, religious understandings and guidance occupy a central role in predominantly Muslim countries such as KSA. Lack of knowledge about the religious views on organ donation in general, and posthumous organ donation in particular, acts as an important barrier to translating positive attitudes into actual donation behavior (6). This highlights the critical role that religious authorities and official religious bodies can play in facilitating the organ donation process, for example, by providing clear, widely disseminated guidance on the permissibility and values of organ donation.

Multiple, complex factors play a significant role in influencing organ donation rates in KSA (2). In a previous study from KSA, psychosocial, cultural, religious, and structural determinants were found to predict organ donation intention in KSA (2). Similar patterns have been reported in other settings in the region, where misconceptions about religion, concerns about body integrity, and mistrust in the healthcare system coexist with generally positive attitudes toward the idea of helping others through donation (3, 7, 8). Although multiple previous studies have shown that the population of KSA generally understands and accepts the concept of organ donation and often expresses a willingness to donate, there are persisting concerns among a significant proportion of the population about various aspects of the organ donation process that act as a barrier to translating willingness into behavioral action regarding organ donation (5). These concerns include fear of body disfigurement, doubts about the fairness of organ allocation, and uncertainty about the quality of medical care for registered donors (2, 3, 5, 7, 8). Moreover, public awareness about the effectiveness of organ donation in transforming the lives of individuals with end-stage organ failure, along with knowledge about the organ donation process, also remains an important area that needs improvement, as knowledge has been found to be an important predictor of organ donation decision-making (9, 10).

Although there are some studies exploring certain aspects of public perception and attitudes toward organ donation in KSA (5, 6), there are limited recent comprehensive studies on the knowledge, attitudes, beliefs, and intentions of the general Saudi population regarding organ donation and transplantation. There is a need for such up-to-date and nationally representative information for healthcare strategists and policymakers to respond effectively to the existing gap between organ supply and demand. Hence, the current study aims to explore knowledge, attitudes, beliefs, and intentions concerning organ donation within the general population of KSA and their associations with sociodemographic factors.

An earlier publication by the author applied the Theory of Planned Behavior (TPB) framework to test specific hypotheses regarding how behavioral, normative, and control beliefs relate to the intention to donate organs in Saudi Arabia (2). That study primarily focused on validating the TPB model and identifying which belief domains were statistically associated with definite donation intention. In contrast, the present analysis adopts a broader, more descriptive perspective: it provides a comprehensive profile of public knowledge, attitudes, beliefs, and behavioral intentions toward organ donation in the general Saudi population, examines how these domains vary across key sociodemographic groups, and derives policy-relevant insights for health strategists and policymakers seeking to address the persistent gap between organ supply and demand in the Kingdom.

The specific hypotheses of the present study posit that elevated levels of knowledge and more favorable attitudes will be correlated with an increased willingness to contribute, and these dimensions will diverge systematically across various sociodemographic groups (such as age, gender, education, income, and nationality), thereby identifying subpopulations that may derive the greatest benefit from targeted interventions.

## Methods

### Study design and setting

This was a cross-sectional survey study conducted among the residents of KSA. The study used an online, self-administered questionnaire designed to assess public knowledge, attitudes, beliefs, and intentions regarding organ donation. The questionnaire was grounded in the TPB model, which posits that behavioral intentions are shaped by three core constructs: behavioral beliefs (expected outcomes of the behavior), normative beliefs (perceived social pressure and expectations), and control beliefs (perceived facilitators and barriers to performing the behavior) (2). Data collection was carried out online between June and December 2022, enabling broad geographic reach across KSA.

### Participants and sampling

Inclusion criteria were:

(1) Residence in the Kingdom of Saudi Arabia,
(2) Age 16 years or older,
(3) Ability to read and understand Arabic, and
(4) Willingness to participate as indicated by completion of the online questionnaire after viewing the study information.

Exclusion criteria included incomplete questionnaires with fewer than 80% of items answered.

**Figure 1:**
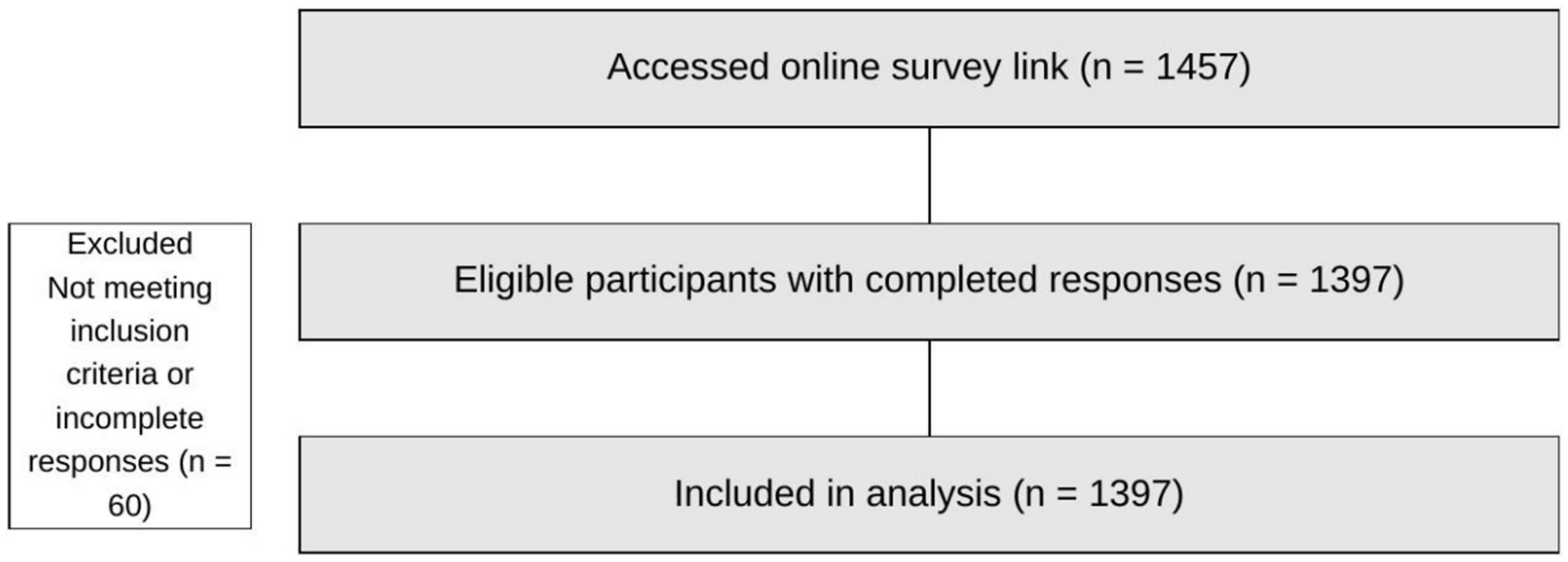
Participant flow diagram.

A convenience sampling technique was employed to recruit participants. The survey instrument was prepared in Google™ Forms and was disseminated via institutional and other community networks. A total of 1,397 valid responses were included in the study (Figure 1).

This online distribution strategy, particularly through institutional networks, may have contributed to an overrepresentation of younger, more educated, and urban respondents; this potential selection bias is acknowledged in the limitations.

No formal *a priori* sample size calculation was performed; given the exploratory nature of the study, our aim was to maximise participation within the data collection period to enhance the precision of estimates and the power to detect associations.

### Instrument

The study questionnaire contained three parts. The first part captured sociodemographic variables such as age, gender, nationality, religion, marital status, education, occupation, average monthly income, and current location. The second part explored general awareness and knowledge regarding various aspects of organ donation, and the final part was adapted from a validated instrument used to investigate beliefs and intentions regarding organ donation in Qatar, which is based on the TPB model (8). The questionnaire captured attitudes (overall evaluations of organ donation), normative beliefs (perceptions of approval or disapproval from important others), control beliefs (perceived facilitators and barriers), and the intention to donate. The items pertaining to the TPB were evaluated on a five-point Likert scale, ranging from strongly agree to strongly disagree, with a neutral option included. Questions regarding willingness to register as an organ donor were asked only of participants who reported not being currently registered as organ donors (*n* = 1,029), while all participants (*n* = 1,397) were asked about their current registration status.

### Ethics

The study’s purpose and consent statement were presented on the survey landing page, and participants could only proceed if they provided informed electronic consent. No personally identifying information was collected. The study protocol received approval from the institutional ethics committee (UJ-REC-234) and complied with the principles of the Declaration of Helsinki.

### Statistical analysis

Data were analyzed using jamovi (The jamovi project; version 2.x). Descriptive statistics summarized participant characteristics and study variables as frequencies and percentages for categorical data, and as means with standard deviations for continuous variables. Associations between willingness to register as an organ donor and sociodemographic variables, knowldege, attitude, and beliefs were examined using chi-square tests for categorical variables and independent-samples t-tests and one-way ANOVA for continuous variables. All tests were two-sided and a two-sided *p*-value < 0.05 was considered statistically significant.

## Results

### Association between willingness to register as an organ donor and sociodemographic variables

Indecision regarding registering as an organ donor was the predominant response across all sociodemographic subgroups. Females reported significantly higher willingness to register than males (10.4% vs. 8.7%) and slightly lower refusal (10.2% vs. 13.6%) (*χ*^2^ = 10.3, *p* = 0.006). There was a statistically significant association between willingness to register as an organ donor and nationality, with Saudi nationals showing a higher willingness to register as an organ donor (*χ*^2^ = 13.8, *p* < 0.001). There was a statistically significant association between occupation and willingness, with non-working respondents showing lower willingness to register as an organ donor (*χ*^2^ = 20.3, *p* = 0.009). Income level also showed a significant association with willingness, with higher-income groups more often undecided (*χ*^2^ = 17.3, *p* = 0.008). There was no significant association between religion, marital status, education level, or location and willingness to register as an organ donor. Mean age did not differ significantly across willingness groups (one-way ANOVA, *F* = 1.13, *p* = 0.32), indicating no association between age and willingness to register as an organ donor. Detailed sociodemographic characteristics and their association with willingness to register are presented in Table 1.

**Table 1:** Association between willingness to register as an organ/tissue donor and sociodemographic variables.

Variable	Are you willing to register as an organ/tissue donor in Saudi Arabia (*n* = 1,029)*				
	Yes	No	Not decided	Chi-square value	*p*-value
Gender					
Male	90 (8.7%)	140 (13.6%)	266 (25.9%)	10.3	0.006
Female	107 (10.4%)	105 (10.2%)	321 (31.2%)		
Nationality					
Saudi	163 (15.8%)	223 (21.7%)	539 (52.4%)	13.8	<0.001
Non-Saudi	34 (3.3%)	22 (2.1%)	48 (4.7%)		
Religion					
Muslim	195 (19%)	242 (23.5%)	573 (55.7%)	3.54	0.472
Christian	0	1 (0.1%)	7 (0.7%)		
Other	2 (0.2%)	2 (0.2%)	7 (0.7%)		
Marital status					
Married	67 (8.5%)	129 (12.5%)	295 (28.7%)	6.50	0.165
Unmarried	104 (10.1%)	105 (10.2%)	278 (27%)		
Others	6 (0.6%)	11 (1.1%)	14 (1.4%)		
Education					
High school	26 (2.5%)	37 (3.6%)	77 (7.5%)	2.78	0.595
Bachelor	126 (12.2%)	165 (16%)	401 (39%)		
Master	45 (4.4%)	43 (4.2%)	109 (10.6%)		
Occupation					
Self-employed	12 (1.2%)	9 (0.9%)	16 (1.6%)	20.3	0.009
Private sector	55 (5.3%)	53 (5.2%)	129 (12.5%)		
Government	54 (5.2%)	57 (5.5%)	151 (14.7%)		
Not working	64 (6.2%)	117 (11.4%)	275 (26.7%)		
Others	12 (1.2%)	9 (0.9%)	16 (1.6%)		
Income (SR)					
<5,000	72 (7%)	105 (10.2%)	246 (23.9%)	17.3	0.008
5,001–10,000	40 (3.9%)	35 (3.4%)	119 (11.6%)		
10,001–15,000	26 (2.5%)	55 (5.4%)	85 (8.3%)		
>15,000	59 (5.7%)	50 (4.9%)	136 (13.2%)		
Region					
Central	23 (2.2%)	41 (4%)	70 (6.8%)	7.40	0.494
Western	131 (12.7%)	157 (15.3%)	380 (36.9%)		
Eastern	27 (2.6%)	27 (2.6%)	81 (7.9%)		
Northern	4 (0.4%)	8 (0.8%)	26 (2.5%)		
Southern	12 (1.2%)	12 (1.2%)	30 (2.9%)		

*Questions about willingness to register were asked only of participants who were not already registered organ donors (*n* = 1,029 of 1,397 total respondents).

Values are presented as *n* (%) for categorical variables.

### Knowledge about organ donation

A majority of the study participants reported a good baseline understanding of organ donation. 83.6% correctly responded that organ donation means the transfer of an organ from deceased or living donors. 56.2% of the study participants could correctly identify brain death as a state with a beating heart supported by ventilation. 83.7% were aware of the Saudi donor registry, and 60.6% believed that enrollment for organ donation is possible from 18 years of age and above. 68.6% reported that their religion permits organ donation, and 25.1% were unsure about religious permissibility for organ donation. Around 35% knew someone who had donated an organ.

89% knew that one can donate part of their liver to a family member, and 50.5% did not consider this a health risk. 84.0% also knew that it is possible to donate a kidney during one’s lifetime, and 55.6% believed that this is safe.

90.4% knew that organ trade is strictly prohibited in KSA. 84.8% knew that organ transplant facilities are provided free of charge to Saudi citizens. 88.9% of respondents knew that deceased-donor organs are allocated to the first individual on the waiting list, and 97.2% knew that exerting pressure on a deceased donor’s family or a living donor is strictly prohibited. The overall distribution of knowledge items is summarized in Table 2.

**Table 2:** Knowledge about organ donation.

Variable	Response	*n* (%)
What does organ/tissue donation mean to you?	Transfer of an organ or tissue from a deceased body to a patient in need.	161 (11.5%)
	Transfer of an organ or tissue from a living donor to a patient in need.	68 (4.9%)
	All of the above	1,168 (83.6%)
What is the meaning of death in the term “donation after death”?	The heart is not beating, and there is no breathing.	431 (30.9%)
	Brain death in which the heart is beating with the help of a ventilator to keep breathing.	785 (56.2%)
	Do not know.	181 (12.9%)
There is a donor registry in Saudi Arabia.	Yes	1,169 (83.7%)
	No	114 (8.2%)
	Partially	114 (8.1%)
At what age may an individual enroll in organ donation?	At any age	149 (10.7%)
	18 years and above	846 (60.6%)
	Do not know	402 (28.8%)
Does your religion allow organ donation?	Yes	959 (68.6%)
	No	88 (6.3%)
	Do not know	350 (25.1%)
Do you know anyone who has donated an organ?	Yes	498 (35.6%)
	No	899 (64.4%)
Do you believe that throughout one’s lifetime, an individual can donate a portion of their liver to a family member?	Yes	1,244 (89%)
	No	153 (11%)
Do you think that donating a part of your liver is a risk to your health?	Yes	121 (8.7%)
	No	706 (50.5%)
	Maybe	448 (32.1%)
	Do not know	122 (8.7%)
Do you believe that it is possible for you to donate one of your kidneys during your lifetime to another individual?	Yes	1,173 (84%)
	No	224 (16%)
Do you think that donating a kidney is safe?	Yes	777 (55.6%)
	No	109 (7.8%)
	Maybe	433 (31%)
	Do not know	78 (5.6%)
Do you know that the trade of human organs is strictly prohibited?	Yes	1,263 (90.4%)
	No	134 (9.6%)
Do you know that the government provides free access to transplant facilities for all Saudi citizens equally?	Yes	1,185 (84.8%)
	No	212 (15.2%)
Do you know that organs donated by deceased individuals are allocated to the first person on the waiting list?	Yes	1,242 (88.9%)
	No	155 (11.1%)
Do you know that it is strictly prohibited to exert pressure on the family of a deceased donor or on a living donor to compel them to donate?	Yes	1,358 (97.2%)
	No	39 (2.8%)

Values are presented as *n* (%) for categorical variables.

### Attitudes toward organ donation

The study results showed that attitudes toward organ donation were generally favorable. A majority of the study population agreed that organ donation is good and should be promoted (54.7% strongly agree, 24.1% agree), and that registering as an organ donor could save a life (67.6% strongly agree, 23.8% agree). However, only 44.3% agreed with automatic enrollment with an opt-out option for the population of KSA.

Willingness to donate an organ increased if participants were assured of no family objection (60.9%), if provided more information about transplantation (68.4%), and if given more information on religious viewpoints (70.1%). Similarly, knowing how to register increased willingness (63.2%). The results are summarized in Table 3.

**Table 3:** Attitudes toward organ donation.

Variables	Response	*n* (%)
Organ donation is a good thing and should be promoted.	Strongly agree	764 (54.7%)
	Agree	337 (24.1%)
	Neither agree nor disagree	256 (18.3%)
	Disagree	23 (1.6%)
	Strongly disagree	17 (1.2%)
Registering as an organ donor could save somebody’s life	Strongly agree	945 (67.6%)
	Agree	332 (23.8%)
	Neither agree nor disagree	100 (7.2%)
	Disagree	9 (0.6%)
	Strongly disagree	11 (0.8%)
Saudi and non-Saudi residents alike should be automatically enrolled in the organ donor registry, with the option to decline should they so choose.	Strongly agree	391 (28%)
	Agree	228 (16.3%)
	Neither agree nor disagree	294 (21%)
	Disagree	237 (17%)
	Strongly disagree	247 (17.7%)
I am willing to donate if I know that my family would have no objection to allowing the donation of my organs at the time of my death	Strongly agree	524 (37.5%)
	Agree	327 (23.4%)
	Neither agree nor disagree	335 (24%)
	Disagree	108 (7.7%)
	Strongly disagree	103 (7.4%)
I am willing to donate if I know more about what organ transplant is and how it is done.	Strongly agree	523 (37.4%)
	Agree	433 (31%)
	Neither agree nor disagree	289 (20.7%)
	Disagree	87 (6.2%)
	Strongly disagree	65 (4.7%)
I am willing to donate if more information is available about the viewpoint of my religion with regard to organ donation.	Strongly agree	589 (42.2%)
	Agree	390 (27.9%)
	Neither agree nor disagree	296 (21.2%)
	Disagree	71 (5.1%)
	Strongly disagree	51 (3.7%)
I am willing to donate if I know how I can register.	Strongly agree	530 (37.9%)
	Agree	354 (25.3%)
	Neither agree nor disagree	344 (24.6%)
	Disagree	94 (6.7%)
	Strongly disagree	75 (5.4%)

Values are presented as *n* (%) for categorical variables.

### Beliefs about organ donation

This study also showed that beliefs regarding organ donation were generally favorable among the study participants. 79.5% believed that donation would be rewarded by God. Only 25.0% believed that registered donors receive compromised care, and only 29.0% believed that organ donation can cause potential body disfigurement. 67.6% believed that social support for families could increase donation.

Administrative barriers were not widely endorsed, with only 16.1% agreeing that the organ registration process is time-consuming, and 37.1% agreeing that they may not get answers to all questions during registration. Operative aspects were viewed as a potential deterrent by only 45.9%. Finally, beliefs about post-donation weakness or disability were present among 56.0% of the study participants. The results are summarized in Table 4.

**Table 4:** Beliefs toward organ donation.

Variables	Response	*n* (%)
I think my donation, whether living or after death, is going to impact my life after death in a good way and will be rewarded by God.	Strongly agree	800 (57.3%)
	Agree	310 (22.2%)
	Neither agree nor disagree	229 (16.4%)
	Disagree	33 (2.4%)
	Strongly disagree	25 (1.8%)
In case of an emergency, doctors will not provide enough care if the patient is a registered organ donor.	Strongly agree	163 (11.7%)
	Agree	186 (13.3%)
	Neither agree nor disagree	482 (34.5%)
	Disagree	256 (18.3%)
	Strongly disagree	310 (22.2%)
Organ retrieval processes after death may cause body disfigurement.	Strongly agree	120 (8.6%)
	Agree	285 (20.4%)
	Neither agree nor disagree	537 (38.4%)
	Disagree	274 (19.6%)
	Strongly disagree	181 (13%)
Organ donation will increase if social support is provided to the family (of the deceased), regardless of whether they donate or not	Strongly agree	451 (32.3%)
	Agree	493 (35.3%)
	Neither agree nor disagree	362 (25.9%)
	Disagree	48 (3.4%)
	Strongly disagree	43 (3.1%)
Organ donor registration can be a time-consuming process	Strongly agree	56 (4%)
	Agree	169 (12.1%)
	Neither agree nor disagree	534 (38.2%)
	Disagree	351 (25.1%)
	Strongly disagree	287 (20.5%)
While registering for organ donation, you may not get an answer to all your questions.	Strongly agree	148 (10.6%)
	Agree	370 (26.5%)
	Neither agree nor disagree	559 (40%)
	Disagree	212 (15.2%)
	Strongly disagree	108 (7.7%)
The operation procedure for procuring organs is discouraging	Strongly agree	217 (15.5%)
	Agree	425 (30.4%)
	Neither agree nor disagree	420 (30.1%)
	Disagree	201 (14.4%)
	Strongly disagree	134 (9.6%)
You are worried that organ donation might leave you weak and disabled.	Strongly agree	273 (19.5%)
	Agree	510 (36.5%)
	Neither agree nor disagree	310 (22.2%)
	Disagree	197 (14.1%)
	Strongly disagree	107 (7.7%)

Values are presented as *n* (%) for categorical variables.

### Association between willingness to register as an organ donor and knowledge, attitude, and belief variables

This study found that willingness to register as an organ donor was significantly associated with various beliefs, attitudes, and knowledge variables. Within beliefs, study participants who believed that donation positively affects life after death and would be rewarded by God were more willing to register (*χ*^2^ = 325, df = 8, *p* < 0.001). Beliefs about poor medical care for registered donors (*χ*^2^ = 40.6, df = 8, *p* < 0.001) and potential bodily deformity following donation (*χ*^2^ = 57.6, df = 8, *p* < 0.001) were also significantly related to willingness. Agreement with the statement that social support for families would increase donation showed a strong association with willingness (*χ*^2^ = 134, df = 8, *p* < 0.001), while viewing registration as time-consuming showed a smaller yet significant association (*χ*^2^ = 16.4, df = 8, *p* = 0.037). Beliefs that procurement procedures are discouraging (*χ*^2^ = 49.7, df = 8, *p* < 0.001) and worries about becoming weak or disabled after donation (*χ*^2^ = 58.2, df = 8, *p* < 0.001) were likewise significantly associated with willingness.

Agreement that organ donation is good and should be promoted (*χ*^2^ = 285, df = 8, *p* < 0.001) and that registering could save a life (*χ*^2^ = 192, df = 8, *p* < 0.001) were significantly associated with willingness. Support for automatic enrollment with opt-out (*χ*^2^ = 158, df = 8, *p* < 0.001) and conditional willingness contingent on family approval (*χ*^2^ = 499, df = 8, *p* < 0.001), greater understanding of transplantation (*χ*^2^ = 489, df = 8, *p* < 0.001), clearer religious guidance (*χ*^2^ = 391, df = 8, *p* < 0.001), and knowing how to register (*χ*^2^ = 547, df = 8, *p* < 0.001) were each significantly associated. Knowledge items were also significantly related to willingness, including knowing the minimum age to enroll (*χ*^2^ = 10.6, df = 4, *p* = 0.031) and religious permissibility (*χ*^2^ = 186, df = 8, *p* < 0.001). Awareness of living donation possibilities and risks—partial liver donation to a family member (*χ*^2^ = 8.21, df = 2, *p* = 0.016), perceived risk of partial liver donation (*χ*^2^ = 68.6, df = 6, *p* < 0.001), feasibility of living kidney donation (*χ*^2^ = 58.2, df = 2, *p* < 0.001), and perceived safety of kidney donation (*χ*^2^ = 91.6, df = 6, *p* < 0.001)—showed consistent associations. System-level knowledge was likewise relevant: awareness of free access to transplant services for Saudi citizens (*χ*^2^ = 18.6, df = 2, *p* < 0.001), allocation of deceased-donor organs by waitlist order (*χ*^2^ = 9.30, df = 2, *p* = 0.010), and the prohibition of coercion (*χ*^2^ = 6.30, df = 2, *p* = 0.040) were all significant. The results are summarized in Table 5.

**Table 5:** Association between knowledge, attitude, and belief items and willingness to register as an organ/tissue donor in Saudi Arabia (Chi-square test).

Variables	Willingness to register as an organ/tissue donor in Saudi Arabia		
	*χ* ^2^	df	*p*-value
Knowledge about organ donation			
What does organ/tissue donation mean to you?	1.21	4	0.877
What is the meaning of death in the term “donation after death”?	3.34	6	0.765
There is a donor registry in Saudi Arabia.	8.74	6	0.189
At what age may an individual enroll in organ donation?	10.6	4	0.031
Does your religion allow organ donation?	186	8	<0.001
Do you know anyone who has donated an organ?	17.1	2	<0.001
Do you believe that throughout one’s lifetime, an individual can donate a portion of their liver to a family member?	8.21	2	0.016
Do you think that donating a part of your liver is a risk to your health?	68.6	6	<0.001
Do you believe that it is possible for you to donate one of your kidneys during your lifetime to another individual?	58.2	2	<0.001
Do you think that donating a kidney is safe?	91.6	6	<0.001
Do you know that the trade of human organs is strictly prohibited?	1.78	2	0.411
Do you know that the government provides free access to transplant facilities for all Saudi citizens equally?	18.6	2	<0.001
Do you know that organs donated by deceased individuals are allocated to the first person on the waiting list?	9.30	2	0.010
Do you know that it is strictly prohibited to exert pressure on the family of a deceased donor or on a living donor to compel them to donate?	6.30	2	0.043
Attitudes toward organ donation			
Organ donation is a good thing and should be promoted.	285	8	<0.001
Registering as an organ donor could save somebody’s life	192	8	<0.001
Saudi and non-Saudi residents alike should be automatically enrolled in the organ donor registry, with the option to decline should they so choose.	158	8	<0.001
I am willing to donate if I know that my family would have no objection to allowing the donation of my organs at the time of my death	499	8	<0.001
I am willing to donate if I know more about what organ transplant is and how it is done.	489	8	<0.001
I am willing to donate if more information is available about the viewpoint of my religion with regard to organ donation.	391	8	<0.001
I am willing to donate if I know how I can register.	547	8	<0.001
Beliefs toward organ donation			
I think my donation, whether living or after death, is going to impact my life after death in a good way and will be rewarded by God.	325	8	<0.001
In case of an emergency, doctors will not provide enough care if the patient is a registered organ donor.	40.6	8	<0.001
Organ retrieval processes after death may cause body disfigurement.	57.6	8	<0.001
Organ donation will increase if social support is provided to the family (of the deceased), regardless of whether they donate or not	134	8	<0.001
Organ donor registration can be a time-consuming process	16.4	8	0.037
While registering for organ donation, you may not get an answer to all your questions.	15.4	8	0.052
The operation procedure for procuring organs is discouraging	49.7	8	<0.001
You are worried that organ donation might leave you weak and disabled.	58.2	8	<0.001

## Discussion

Organ transplantation significantly reduces morbidity and mortality in patients with end-stage organ diseases. However, the availability of donor organs is far less than the ever-increasing need for organ donation. To understand this public health issue and devise tailored solutions, it is crucial to understand public perceptions about organ donation and the factors influencing the decision to become a donor in different countries and cultural contexts. This study aimed to understand the knowledge, attitudes, and beliefs of the Saudi population regarding organ donation and their willingness to register as organ donors.

### Knowledge about organ donation

This study revealed that the knowledge levels of the study population were generally high. Over 80% of the respondents accurately identified organ donation as the transfer of organs or tissues from a deceased or living donor to a patient in need. This level of conceptual understanding is consistent with recent findings from neighboring countries in the region. For example, university staff in Oman demonstrated generally favorable knowledge about organ donation, although with notable gaps in specific domains (11), while a national survey in Tunisia similarly reported good awareness but variable depth of understanding across items (12). An earlier study from Qatar reported that approximately 62.7% of the respondents correctly recognized this concept (13). In contrast, a study from Jordan indicated that over 90% of the respondents accurately identified organ donation (14). Taken together, these data suggest that the Gulf and broader Arab region share a relatively strong baseline understanding of the basic concept of organ donation, with important nuances at item level.

A key knowledge gap in our study concerned brain death. While 56.2% of respondents correctly recognized brain death as a state with a beating heart supported by ventilation, this proportion is lower than that reported in a national survey from the United Arab Emirates, where 88.1% correctly identified the concept (15), and higher than recent findings from Qatar (30.7%) (13). Considering the centrality of brain death to deceased organ donation, this represents a critical weakness. The moderate level of conceptual clarity about brain death in our sample suggests that many individuals may support organ donation in principle but remain uncertain about when donation is religiously and medically permissible. Given that death is a religiously informed concept, educational efforts that explicitly explain brain death in both medical and religious terms are likely to be particularly impactful.

Other knowledge domains were comparatively strong. Most notably, awareness that pressuring donors is forbidden was very high (94%), indicating that ethical principles related to voluntariness and the prohibition of coercion are well internalized by the public. This suggests that many individuals understand organ donation as an act that should be free, voluntary, and morally safeguarded, which is an important foundation for building trust in the system.

Moreover, in this study, religious beliefs emerged as a facilitator rather than a barrier. 79.5% perceived organ donation as rewarded by God. More than 70% of the respondents also reported that their willingness to donate organs would increase if more information were available about their religion’s stance on organ donation. This underscores the profound influence of religion on decisions regarding organ donation. In Saudi Arabia, where religious authorities have officially recognized brain death and permitted organ donation, it is advisable to engage with religious scholars to provide religion-based education on the permissibility of organ donation (16). This educational approach can motivate individuals to register as organ donors.

The study participants demonstrated a strong understanding of organ trade, knowing it is prohibited, and were aware of equal and free access to transplant services in Saudi Arabia. These findings are encouraging and can be attributed to the long-standing national policy and infrastructure development in the organ transplantation sector. However, our study also identified some knowledge gaps. Approximately 40.8% reported that donating a part of their liver is or may be a risk to their health, while around 38.8% reported that donating a kidney is not or may not be safe. Such concerns are potent barriers to making the decision to become an organ donor even when general attitudes are positive. Clear, repeated communication from clinical authorities about surgical safety, donor selection, and follow-up could help convert positive sentiment into concrete willingness and registration.

### Attitudes toward organ donation

Overall, respondents’ attitudes toward organ donation were favorable at a general level but more ambivalent when translated into concrete behavioral intentions such as registration. Many participants agreed that organ donation is good and should be promoted and that registering could save lives, yet a large proportion remained undecided about actually registering as donors. This pattern—positive attitudes but hesitant action—suggests that attitudinal support alone is not sufficient to drive registration behavior.

Certain attitudinal strengths stood out. Respondents expressed high levels of support for equitable allocation of organs and recognition of the importance of transplant services, indicating a broadly pro-donation normative climate. At the same time, concerns about potential health risks of living donation, particularly partial liver donation, and anxieties about possible harm or complications for the donor, were prominent. These concerns appear to weaken willingness to commit to donation, despite generally positive attitudes toward the idea of helping others.

In the Saudi context, addressing these attitudinal barriers will require clear, evidence-based information about the medical risks and safeguards associated with living and deceased donation, including perioperative care, long-term follow-up, and donor protection mechanisms. Providing testimonials from living donors and transplant recipients, and transparent statistics on donor safety, may help convert favorable attitudes into more confident decisions to register.

### Beliefs, religion, and social norms

Religious beliefs emerged in this study as a facilitator rather than a barrier. 79.5% of respondents perceived organ donation as rewarded by God, and 68.6% reported that their religion permits organ donation, with an additional 25.1% uncertain about religious permissibility. This suggests that explicit opposition to organ donation on religious grounds is relatively uncommon; instead, uncertainty or incomplete knowledge of religious rulings is more salient. More than 70% of respondents also reported that their willingness to donate organs would increase if more information were available about their religion’s stance on organ donation. This underscores the profound influence of religion on organ donation decisions.

Social and familial beliefs also played a key role. Many respondents indicated that family approval is essential, and conditional willingness contingent on family support was strongly associated with willingness to register. This suggests that subjective norms—especially perceived expectations from family members—are among the most critical determinants of behavior in the Saudi context, consistent with the Theory of Planned Behavior (17). Encouraging family-level conversations about organ donation is therefore likely to be as important as individual-level education (18). Pragmatic educational interventions regarding various aspects of organ donation, rather than purely theoretical interventions, can also be more effective in converting positive attitudes into behavioral responses (19). Additionally, making the organ registration process easy and accessible can motivate individuals to register as donors.

### Willingness to register

In the present study, only 19.1% of the participants expressed willingness to register as organ or tissue donors, which aligns with previous research. A recent study conducted among the general population in Saudi Arabia revealed that only 25% expressed a willingness to donate their kidneys, while only 4% were already registered as donors (20). Another study from Qatar indicated that only 29.8% were willing to register as organ or tissue donors (10). This result emphasizes the need for targeted interventions to translate positive attitudes and beliefs into behavioral responses. In this context, the positive associations can offer further direction for targeting. This study revealed positive associations between willingness to register as an organ or tissue donor and gender, nationality, occupation, and income. The higher willingness among women compared to men aligns with previous studies, necessitating gender-sensitive messaging and community engagement to translate this willingness into action across both groups (21–23). A recent study from Saudi Arabia also reported that females and diploma/graduation-level education were significantly associated with opt-out organ donation registration support (24). Additionally, the higher indecision among those not employed and in lower income brackets suggests that tailored outreach should focus on financial protections, workplace reintegration after living donation, and clarity about the absence of costs for recipients. In this context, outreach in Saudi Arabia should also clearly communicate the existing donor support framework. The Saudi Center for Organ Transplantation describes several benefits for organ donors, including eligibility for the King Abdulaziz Medal (Third Class), financial compensation for families of deceased donors, discounted airfare on Saudi Arabian Airlines for kidney and liver donors, and donor identification cards intended to facilitate access to services (25). Despite these provisions, our respondents’ concerns about financial burden suggest that awareness and perceived adequacy of such protections remain limited, particularly among non-working and lower-income groups. International policy analyses further highlight that living donors can face substantial out-of-pocket expenses and lost income, and propose strategies such as reimbursement of travel and accommodation, wage replacement during evaluation and recovery, and stronger legal protections against employment or insurance discrimination to move toward financial neutrality for donors (26, 27). Adapting and clearly communicating similar principles within the Saudi context—while building on current donor rights—could help reduce perceived financial risk and support more equitable willingness to donate.

### Targeted interventions and policy implications

The convergence of relatively high knowledge, strong religious endorsement, and a generally supportive normative climate—alongside persistent ambivalence and specific misconceptions—points toward several targeted intervention strategies in KSA.

First and foremost, awareness building measures using educational content should prioritize: (a) clarifying the concept of brain death in both medical and religious language; (b) explaining the safety, eligibility criteria, and follow-up care for living donors; (c) correcting misconceptions about the minimum age for registration and the legal protections against coercion; and (d) outlining the practical steps required to register as a donor (e.g., via national registries and digital government platforms).

Second, community channels should be carefully selected to match the sociocultural context. Potentially effective channels in Saudi Arabia include mosque-based sermons and study circles led by imams and religious scholars, collaborations with the Saudi Center for Organ Transplantation, targeted campaigns in schools and universities, and the integration of organ donation messages into widely used digital platforms (e.g., government health applications and e-services portals). Utilizing social media influencers who are perceived as credible and value-aligned may further extend reach among younger audiences.

Third, the engagement of religious scholars should be structured rather than *ad hoc*. This could involve jointly developed educational materials (e.g., short videos, infographics, and khutba templates) that clearly state the religious permissibility and potential reward of organ donation, address common fears about body integrity and burial rites, and explicitly affirm that coercion is prohibited. Public endorsements from high-profile religious leaders, combined with opportunities for community members to ask questions in open forums, may help resolve residual doubts and increase confidence in registration.

Finally, policies that enhance donor protections and financial security—such as clear coverage of donor medical costs, assurance of job protection during recovery, and transparent communication of legal safeguards—may reduce perceived financial and social risks, thereby supporting a more equitable willingness to donate across income groups.

## Conclusion

This study found that a favorable normative climate regarding organ donation, strong foundational knowledge, and substantial religious endorsement are present among the Saudi public; however, it also uncovered persistent ambivalence and specific misconceptions that likely inhibit actual registration and donation behaviors. Among the three domains examined, foundational knowledge about the general concept of organ donation and ethical safeguards (e.g., prohibition of coercion) appears relatively strong, while attitudes and beliefs related to risk perception, brain death, and family approval show more pronounced weaknesses. In particular, uncertainty about brain death, concerns about the safety of living donation, and the need for explicit family and religious affirmation seem to be the key factors limiting willingness to register.

From a practical perspective, beliefs and subjective norms—especially those shaped by religion and family—may be the most powerful levers for change in the Kingdom of Saudi Arabia, whereas specific aspects of knowledge (e.g., brain death, registration procedures, and donor protections) require targeted strengthening. Sustained, rigorous, and carefully tailored interventions that combine religion-based education, family-focused communication, and clear information about medical and legal protections are imperative to translate positive attitudes and beliefs into a genuine willingness to register as organ donors in Saudi Arabia.

## Data Availability

The raw data supporting the conclusions of this article will be made available by the authors, without undue reservation.
